# Using artificial intelligence to predict patient outcomes from patient-reported outcome measures: a scoping review

**DOI:** 10.1186/s12955-025-02365-z

**Published:** 2025-04-11

**Authors:** Zuzanna Wójcik, Vania Dimitrova, Lorraine Warrington, Galina Velikova, Kate Absolom

**Affiliations:** 1 UKRI Centre for Doctoral Training in Artificial Intelligence for Medical Diagnosis and Care, University of Leeds, Leeds, UK; 2 School of Computer Science, University of Leeds, Leeds, UK; 3 Leeds Institute of Medical Research, University of Leeds, St James’s University Hospital, Leeds, UK; 4 Leeds Cancer Centre, Leeds Teaching Hospitals NHS Trust, Leeds, UK; 5 Leeds Institute of Health Sciences, University of Leeds, Leeds, UK

## Abstract

**Purpose:**

This scoping review aims to identify and summarise artificial intelligence (AI) methods applied to patient-reported outcome measures (PROMs) for prediction of patient outcomes, such as survival, quality of life, or treatment decisions.

**Introduction:**

AI models have been successfully applied to predict outcomes for patients using mainly clinically focused data. However, systematic guidance for utilising AI and PROMs for patient outcome predictions is lacking. This leads to inconsistency of model development and evaluation, limited practical implications, and poor translation to clinical practice.

**Materials and methods:**

This review was conducted across Web of Science, IEEE Xplore, ACM, Digital Library, Cochrane Central Register of Controlled Trials, Medline and Embase databases. Adapted search terms identified published research using AI models with patient-reported data for outcome predictions. Papers using PROMs data as input variables in AI models for prediction of patient outcomes were included.

**Results:**

Three thousand and seventy-seven records were screened, 94 of which were included in the analysis. AI models applied to PROMs data for outcome predictions are most commonly used in orthopaedics and oncology. Poor reporting of model hyperparameters and inconsistent techniques of handling class imbalance and missingness in data were found. The absence of external model validation, participants’ ethnicity information and stakeholders involvement was common.

**Conclusion:**

The results highlight inconsistencies in conducting and reporting of AI research involving PROMs in patients’ outcomes predictions, which reduces the reproducibility of the studies. Recommendations for external validation and stakeholders’ involvement are given to increase the opportunities for applying AI models in clinical practice.

**Supplementary Information:**

The online version contains supplementary material available at 10.1186/s12955-025-02365-z.

## Introduction

Artificial Intelligence (AI) is a field of computer science and engineering which uses computer systems able to mimic intelligent behaviour [1]. AI is known to have potential to improve the effectiveness, accessibility and accuracy of screening, diagnosis and treatment in many areas of health [2, 3]. AI models predicting patient outcomes can achieve high performance, and as a result aid clinical decisions and improve quality of healthcare [3]. AI has been applied to various data types in medicine, using mainly clinical data, such as diagnostic images, genetic data, or brain activity data [4].

While there is a growing attention at patient-reported data in clinical practice and some attempts to use AI models on such data exist [5], systematic guidance on how to apply AI on patient-reported data for outcome predictions is lacking. Patient-reported data can be collected using patient-reported outcome measures (PROMs). These are questionnaires which measure patients’ perception on their health status, without being influenced by clinical opinion [6]. PROMs data can be either standardised and validated tools designed to capture patients’ reports, or any other forms of symptom and quality of life measures [7]. For instance, mobile applications for PROMs collection, have been widely used in healthcare and have potential to improve the quality and personalisation of patient care [8]. The recent systematic evaluation of PROMs in clinical trials of AI health technologies has shown that patients’ perspective is central even in novel technological advancements [9].

Unfortunately, the complexity of PROMs data and limited universal guidelines for AI use in healthcare research [10], can lead to inconsistent reporting of study design and evaluation [11]. Furthermore, studies often lack reproducibility, external validity [12], and generalisability of the results to the clinical context [10]. Inadequate and inconsistent selection of patient-reported input data also introduces a challenge to useful application of patient-centred AI models in healthcare [13]. Additionally, there is a lack of patient and clinician involvement in the process of study design, which plays an important role in addressing bias in AI research for healthcare [14].

There are existing literature reviews exploring AI models applied on PROMs data. For example, a scoping review from 2021 investigated PROMs as standalone input variables in models, however, they did not explore reproducibility and clinical adoption of studies. Moreover, only 2 medically oriented literature databases were searched, while databases from engineering and computer science backgrounds were not considered [5]. Other existing reviews focused on specific healthcare domains (e.g. oncology) and did not investigate all potential applications of using AI and PROMs data. [15–17].

This review aims to address the gap in the literature by investigating AI models used in primary studies for predicting patient outcomes using PROMs. It focuses on methodological rigour of conducting, evaluating and reporting AI research including PROMs as input data. It highlights the importance of ensuring standardised dataset description and justification for chosen methods of model development and evaluation, focusing on clinical relevance. Recommendations for engaging stakeholders, including patients, are suggested.

## Materials and methods

The methodology of this scoping review was based on the Joanna Briggs Institute (JBI) guidance [18]. The review protocol is available on Open Science Framework [19]. The completed PRISMA checklist for scoping reviews [20] is added in the Supplementary Materials Fig. 1 and 2.

### Search strategy

The databases used to search for relevant papers were: Web of Science, IEEE Xplore, ACM Digital Library, Cochrane Central Register of Controlled Trials, Medline and Embase. These databases were selected to include the variety of fields publishing papers on AI in medicine, covering both medical and engineering aspects. The keywords adapted to each database are listed in Supplementary Materials Table 1. Initially, the limited search of Web of Science and Medline was conducted to analyse and approve the keywords. The finalised search of all the records across all the databases was completed on the 7 th November 2023. The reference lists of all relevant reports were also screened. All studies identified through the search strategy were exported to Endnote citation management system.

### Inclusion and exclusion criteria

The inclusion and exclusion criteria followed the Population/Concept/Context (PCC) framework [18] and are described in Table 1. The participants in the papers included in the review were patients, whose symptoms and quality of life data were recorded using various PROMs. These can include mobile applications, or surveys completed either online or in a clinic. The type of data can be collected through either validated and widely used PROMs, or any other patient self-reports. Papers reporting the use of PROMs data as both a predictive and predicted variable were included in the study. If PROMs data were only used as a predicted variable, and not included as inputs, the reference was excluded. The concept was the methods of AI used for predictions of the patients’ outcomes. Papers that explicitly mentioned use of AI or Machine Learning methods were included. Any papers using AI models for purposes other than prediction were excluded. Papers that reported prediction of patient outcomes in the healthcare context were included in the analysis. These outcomes should belong to the categories of patient-related outcomes identified by Kersting et al. (2020) [21], presented in Table 1. The broad understanding of healthcare context allowed focusing on the AI used for various medical reasons.

**Table 1:** Inclusion and Exclusion criteria for the study selection

Category	Inclusion criteria	Exclusion criteria
Input variables	Data reported by patients using standardised PROMs; electronic data collection designed for self-reporting of symptoms or clinical outcomes. These can be used with combination of different non-patient reported data	Only not patient-reported data, e.g., data reported through a clinician, recorded, written down during an appointment, data reported on online forums/social media, or data from physiological measurements
Output variables	Any data describing patient outcomes: sleep behaviour, coping and self-efficacy, healthcare utilisation, body image perception, function, communication skills, reliability of diagnosis and therapy, optimal support, confidence in therapy, satisfaction, cognitive performance, treatment decision, disease control, daily activities, reoperation, mental health, quality of life, mobility, co-morbidities, pain, survival, adverse events, and symptoms	Output that does not relate to health or healthcare, or papers which did not aim to predict outcomes
Models	Machine learning or deep learning predictive models	Statistical models (e.g., only regression analysis)
Paper type	Primary research reported in English language	Abstracts only, theses, dissertations, letters to editors, guidelines, commentaries, introductions, papers published not in English, or review papers

### Review process

All duplicates found in the databases were removed automatically in Endnote. Titles and abstracts of the papers were screened by a researcher and re-selected based on inclusion and exclusion criteria, presented in Table 1. Full texts of articles admitted to the study were assessed against the inclusion and exclusion criteria again. The researcher’s approach was validated through a second reviewer, who repeated scanning through 10% of abstracts, selected full texts and compared their results with the first reviewer. The validation showed high consistency between the reviewers’ decisions, as out of 218 validated papers, 184 (84.4%) were consistently selected or rejected. Therefore, no further validation was performed.

### Data extraction and analysis

The data was extracted from all papers selected for this review. Extracted and summarised information for each included paper is presented in Supplementary Materials Tables 1 and 2. A second reviewer extracted data from 10% of admitted papers for the purpose of validation, and the extracted information was compared and agreed between the reviewers. The summary of data was reported in tabular form in Excel spreadsheet and presented in a narrative form in this review.

The extracted and analysed data included:

- Study characteristics (country and year of publication, healthcare domain, input PROMs variables used, types of PROMs, output variable types, and sample sizes)
- Data pre-processing (missingness in the datasets, missing data imputation techniques, class distribution, techniques for handling class imbalance)
- Model development (types of AI models used, frequency of AI models used, AI techniques for addressing temporality in data, hyperparameter tuning)
- Model evaluation (performance metrics used, variable importance, best-performing AI models)
- Clinical relevance and adoption (patients and clinicians involvement in the study design, validation and deployment stage of research, reporting of sociodemographic information)

## Results

**Fig. 1:**
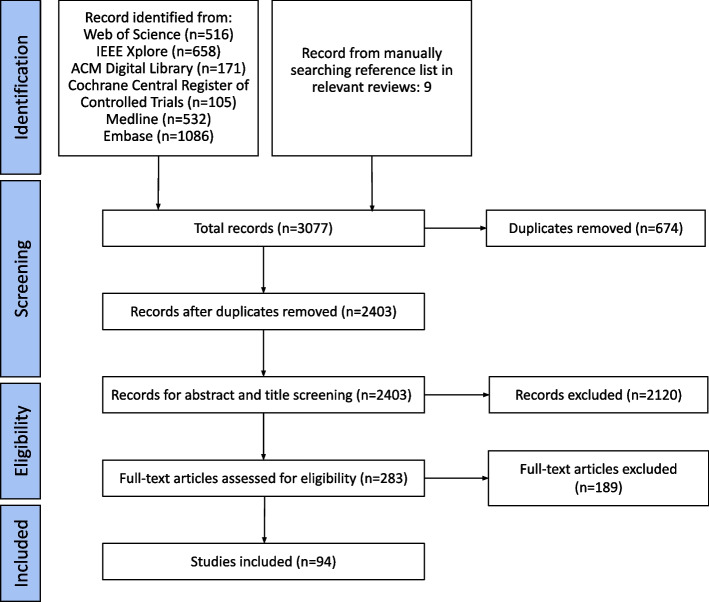
PRISMA flow diagram

Out of 3077 records screened, 94 were selected for analysis in this review. PRISMA diagram [20] (Fig. 1) illustrates the process of paper selection. The reasons for paper exclusions were: no full-text available (38.1%), no PROMs used as input variables (35%), or no AI models used (12%), or methods did not aim to predict patient outcomes (8.75%).

### Study characteristics

**Fig. 2:**
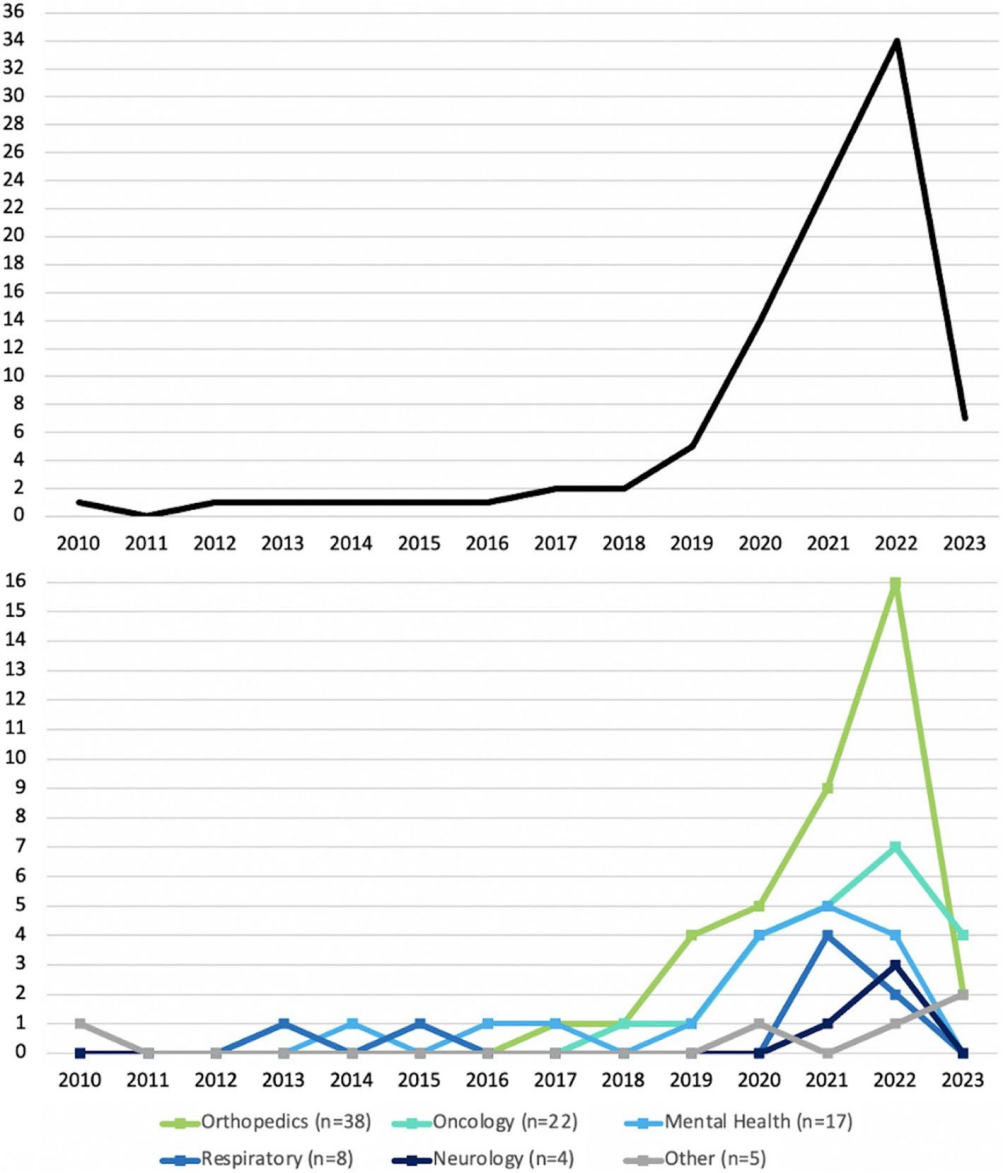
Year of publication of all 94 studies (top figure) and studies based on health domain (bottom figure)

**Fig. 3:**
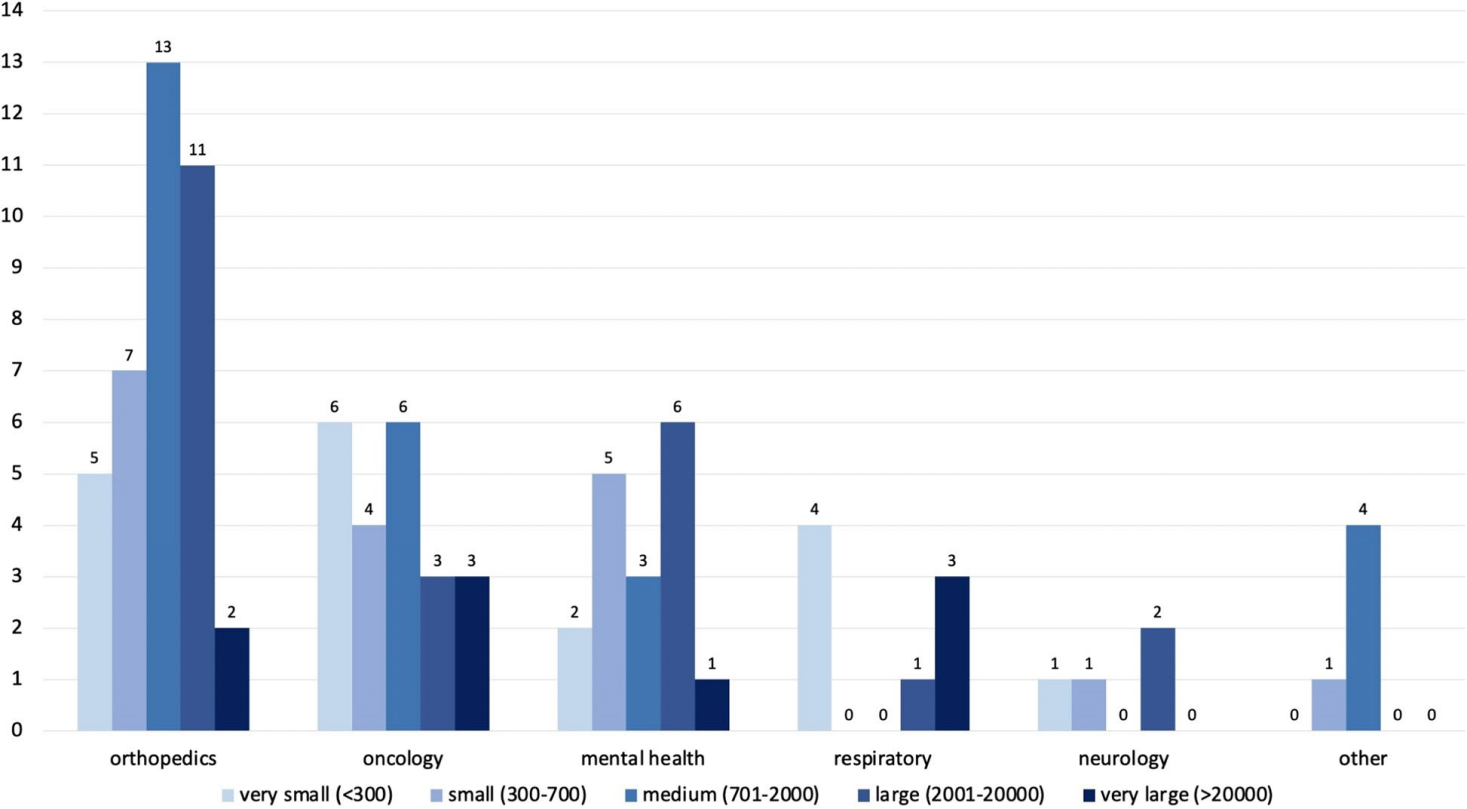
Number of papers categorised based on sample sizes in each healthcare domain

Among the identified studies, 33 (35%) were conducted in USA, 31 (33%) in Europe, 6 (6%) in Canada, 15 (16%) in Asia, 1 (1%) in South America, 1 (1%) in New Zealand, and 1 (1%) in Turkey. Six (6%) studies were conducted internationally (USA and Canada (*n* = 2, 2%); UK and USA (*n* = 2, 2%); Canada and Sweden (*n* = 1, 1%); Europe, US, Australia and Israel (n = 1, 1%)). Identified papers focused on orthopedics (*n* = 38, 40%), oncology (*n* = 22, 23%), mental health (*n* = 17, 18%), respiratory (*n* = 8, 9%), neurology (*n* = 4, 4%) and other domains (*n* = 5, 5%), which appeared only once: hearing, endometriosis, palliative care, sub-health state, and cardiovascular. The studies were published between 2010 and 2023 (Fig. 2). The data were obtained either from existing registry/database (*n* = 44, 47%), or pre-existing or current research studies (*n* = 47, 50%, not reported: *n* = 3, 3%).The self-reported input variables were combined with clinical and demographic data (*n* = 63, 67%), only demographic data (*n* = 14, 15%), only clinical data (*n* = 3, 3%), or other types of data (*n* = 4, 4%), such as wearable, electroencephalographic, bio-mechanical, or family data. Ten studies used only self-reported data for predictions. Most papers (*n* = 63, 67%) were predicting self-reported outcomes, 14 (15%) of which were Minimally Clinically Important Differences (MCID) between pre- and post-clinical event data collection. Other papers used either only objectively measured outcome (*n* = 28, 30%), or a combination of self-reported and objective outcomes (*n* = 3, 3%). Sample sizes of the papers varied from 20 to 1,434,868 (mean = 25,888, median = 1022, 1 st quartile = 429.75, 3rd quartile = 2879.75). The quartiles do not indicate clear boundaries between the data, as there are small differences in the sample sizes. Hence, a boundary-based approach was followed instead of quartile-based: very small (< 300), small (300–700), medium (701–2000), large (2001–20000), and very large (> 20,000), as presented in Fig. 3. The vast majority of studies (*n* = 69, 73%) used condition-specific PROMs, such as orthopedic-specific Knee Injury and Osteoarthritis Outcome Score (KOOS) [22] or cancer-specific EORTC Core Quality of Life Questionnaire (QLQ-C30) [23]. In 31 papers (33%) condition-specific measures with generic questionnaires, for example EuroQol- 5D (EQ- 5D) [24]. Twelve papers (13%) used generic measures only. Out of 81 (86%) papers that reported the types of questionnaires used, 18 (22%) focused on physical health, 11 (14%) on mental health, and 52 (64%) on both.

### Data pre-processing

**Fig. 4:**
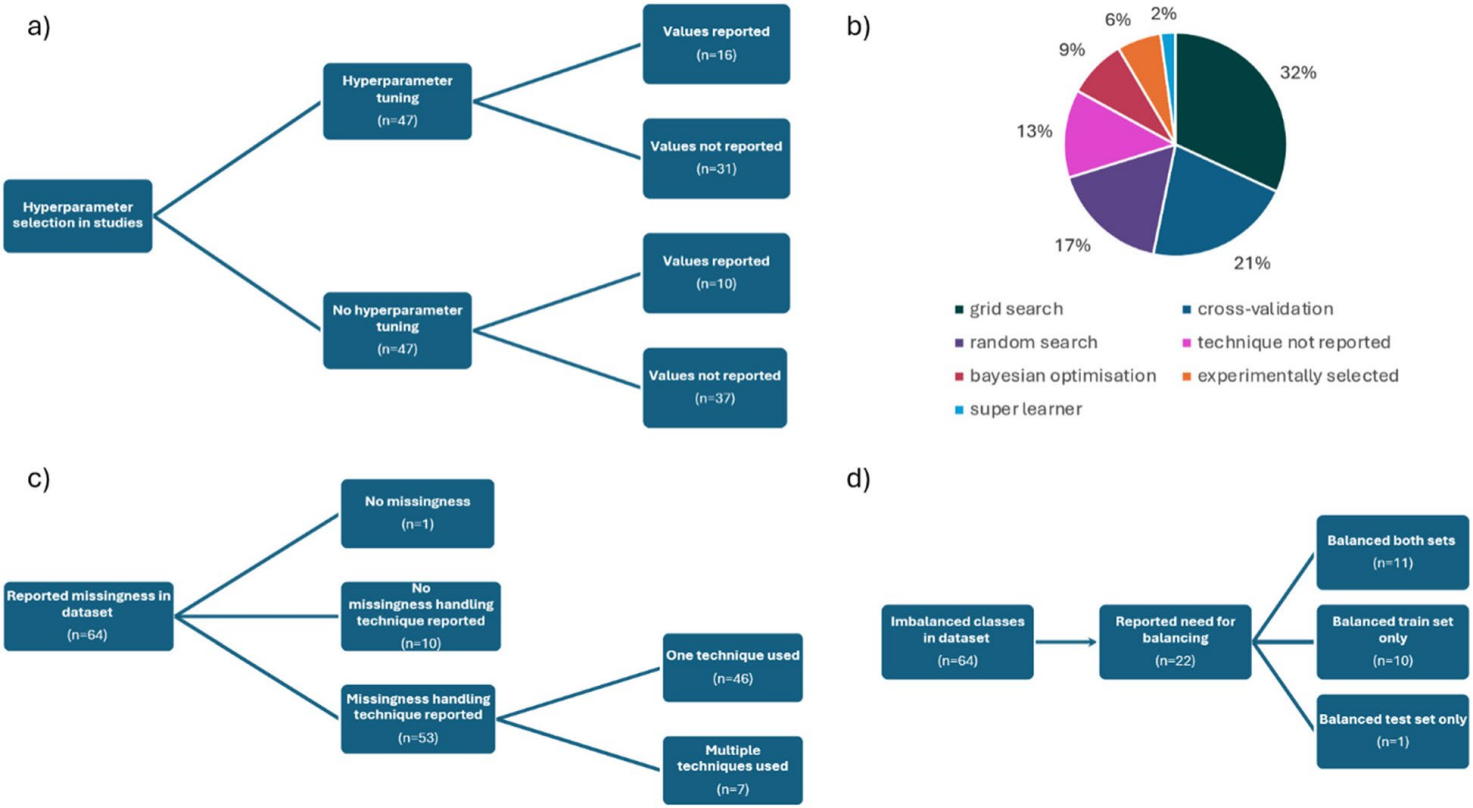
Reporting of pre-processing and model development methods in the studies. Sub-figure **a**) Frequency of hyperparameter tuning and values reporting. Sub-figure **b**) Proportion of hyperparameter tuning techniques. Sub-figure **c**) Missingness reporting and imputation in papers. Sub-figure **d**) Handling class imbalance in studies

Thirty papers did not report the missingness in the dataset. Therefore, it is uncertain if the datasets in these studies did not have any missing data, or if missing data were not disclosed. Out of 64 papers (68%) which reported missing data, only 1 (2%) stated that there was no missingness in the dataset. Ten papers (16%) which reported having missing data did not report how the missingness was handled or addressed. Out of all papers, only 53 (56%) reported the technique for data imputation (Fig. 4c). The 2 most common techniques were complete case analysis (*n* = 16, 30%), and mean/median/mode imputation (*n* = 15, 28%). Most papers (*N* = 89, 95%) used classification as a prediction method. Fourteen (16%) of these did not provide any information about the class distribution. All papers which reported class distribution (*n* = 75, 80%) performed binary classification. Out of these papers, only 11 (15%) had balanced classes (maximum imbalance ratio of 60:40 between the minority and majority class [25]). Sixty-four papers (68%) used dataset with imbalanced classes, 29 (45%) of which did not mention the class imbalance problem. Thirty-five papers (55%) acknowledged the issue but 13 (37%) of them left the data imbalanced. In total, 22 papers (23%) reported the need for balancing the classes, but there was inconsistency in the methods across papers (Fig. 4).

### Model development

**Fig. 5:**
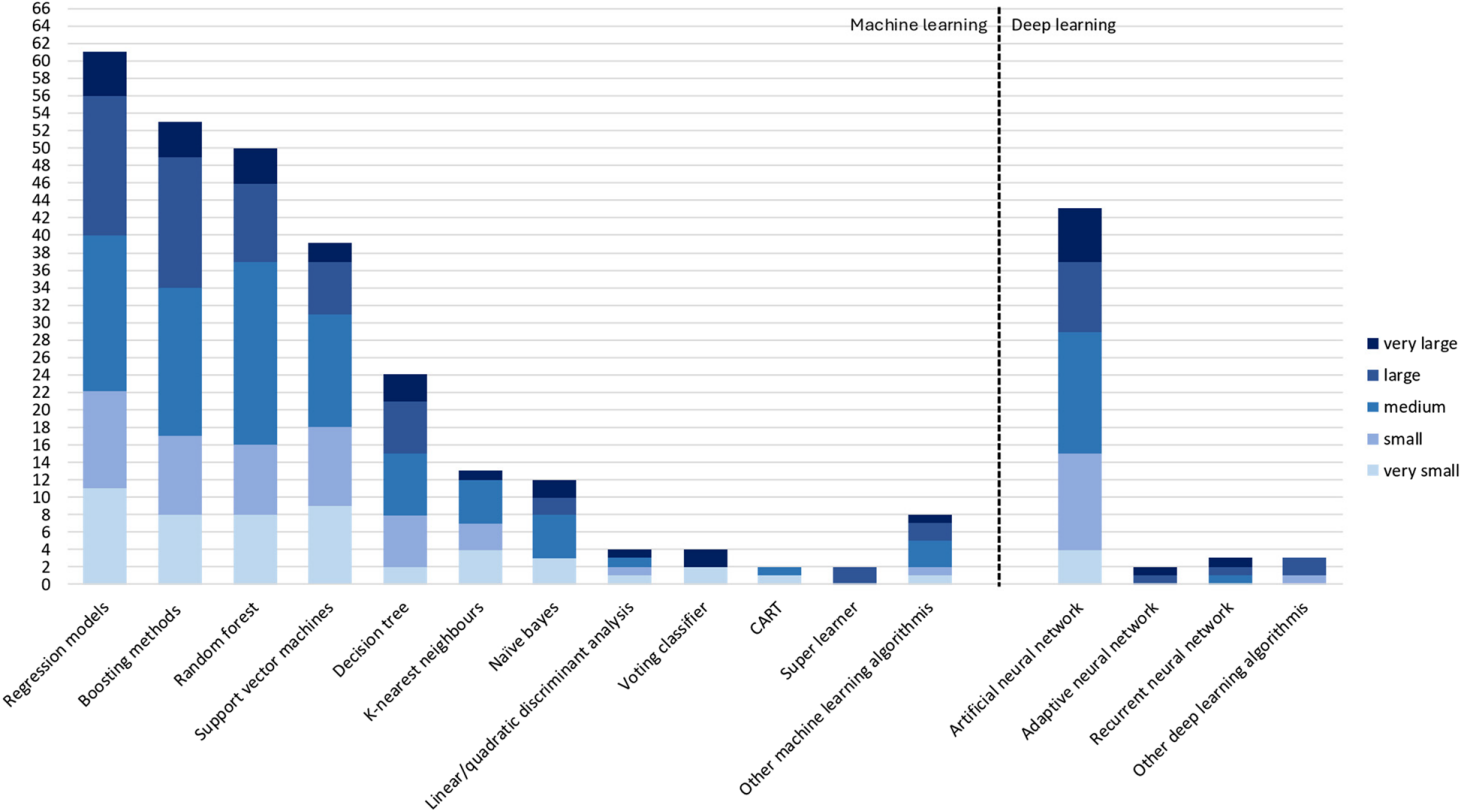
Frequency of algorithms used on datasets with very small (fewer than 300), small (300–700), medium (701–2000), large (2001–20000) and very large (more than 20,000) sample size

Most papers (*n* = 84, 89%) used multiple AI models for outcomes prediction. Forty papers (43%) used only traditional machine learning (ML) models, 5 (5%) only deep learning (DL) models, and 49 (52%) both ML and DL. The most frequently used models were regression models (*n* = 61, 65%), including linear, logistic, ridge and LASSO regressions; boosting methods (*n* = 53, 56%), including adaptive boosting, extreme gradient boosting and gradient boosting machine; random forest (*n* = 50, 53%); artificial neural network (*n* = 43, 46%), including single-or multi-layer perceptrons; and support vector machine (*n* = 39, 41%) (Fig. 5). Most studies (*n* = 74, 81%) applied AI models on data recorded in one time-point. The remaining studies trained their models on data collected in multiple time-points (Table 2). Out of these, 3 studies (3%) reported using models that process the temporal dependencies in the data, such as long-short term memory (LSTM) model [26, 27], and recurrent neural network with gated recurrent units (GRU) [28]. Nine studies (12%) considered temporality through coding it in the feature sets, and 5 papers (7%) did not address temporality at all (Table 3). Half of the papers in this review (*n* = 47, 50%) reported performing hyperparameter tuning, and out of these only 16 (34%) reported used hyperparameters (Fig. 4a)).

**Table 2:** Machine learning and deep learning models used on data collected in one and multiple timepoints, ordered by number of publications

Machine Learning (ML)	One Timepoint	Multiple Timepoints
Regression (*n* = 61)	[29–39] [40–50] [40, 51–60] [61–71] [72–75] [76]	[77–86] [28, 87, 88]
Boosting (*n* = 53)	[31, 32, 36–40, 42, 44, 89, 90] [45, 48, 49, 51–53, 91–94] [40, 56, 58, 60, 62–64, 95–97] [2, 33, 66, 69, 72, 73, 75, 98–100] [70, 74, 101] [76] [102]	[28, 77–79, 81–84, 103, 104]
Random Forest (*n* = 50)	[29–33, 36, 41, 42, 44, 89, 105] [45–48, 50–53, 56, 92, 106] [58, 60, 61, 64, 65, 69, 75, 95, 100, 107, 108] [62, 63, 71, 73, 74, 97, 101]	[26, 28, 77, 78, 80, 87, 88, 104, 109, 110]
Support Vector Machine (*n* = 39)	[29, 31, 32, 34, 37, 41–43, 45, 46, 48] [26, 50–52, 56, 57, 92, 96, 111, 112] [61, 62, 64, 66, 71, 74, 89, 107, 108] [102]	[78, 81, 83, 84, 87, 88, 103, 104]
Decision Tree (*n* = 24)	[37, 39, 41, 57, 92–95, 111, 112] [2, 58, 63, 74, 96, 98, 100, 101, 108]	[77, 79, 84, 103, 109]
K-Nearest-Neighbours (*n* = 13)	[34, 36, 37, 39, 48, 56, 61, 74, 113]	[26, 83, 87, 104]
Na¨ıve Bayes (*n* = 12)	[36, 44, 50, 57, 70, 73, 74, 94, 108]	[26, 104, 109]
Voting Classifier (*n* = 4)	[37, 44]	[28, 82]
Discriminant Analysis (*n* = 4)	[34, 38, 94, 108]	None reported
Classification and Regression Tree (*n* = 2)	[34, 46]	None reported
Super Learner (*n* = 2)	[62, 69]	None reported
Other ML Methods (*n* = 8)	Wide and Deep [49] Stochastic Gradient Descent [61] Bagging [63] Bayesian Updating Algorithm [66] Graphical Gaussian Model [67] Multivariate Adaptive Regression Spline [41]	Hierarchical Gaussian Process [85] Autoregressive Integrated Moving Average [26]
**Deep Learning (DL)**	**One Timepoint**	**Multiple Timepoints**
Multilayer Perceptron (*n* = 43)	[29, 30, 35–37, 39–42, 45, 90] [48, 52–54, 56, 92, 94, 111, 112, 114] [40, 57, 59, 61, 64, 66, 68, 96, 107, 115] [72–75] [76]	[26, 28, 79, 83, 84, 86, 104, 109, 116]
Recurrent Neural Network (RNN) (*n* = 3)	None reported	Long-Short Term Memory [26, 27] RNN with Gated Recurrent Units [28]
Other DL Methods (*n* = 4)	Adaptive Neural Network [36] Stacking Algorithm [57] Bayesian Network Model [117]	Adaptive Neural Network [27]

In the square brackets we list the number of the cited paper, according to the reference lis

**Table 3:** Methods of addressing temporality in time-series data

Level of addressing temporality	Method of addressing temporality	Description of the method	Sample size	Frequency
Addressed by the model	Recurrent Neural Network (RNN)	Data transformed to 3D array and fed in the LSTM model [26] Events encoded by Adaptive Net, pooled by LSTM model [27]	823 9,500	Weekly Irregular^a^
		RNN with GRU, considering each treatment as timestep [28]	105,129	Irregular^a^
Addressed in the features	Measured change	Change of measurement from baseline [84] Change in mean measurements from baseline [88] Mean daily change from the 24-h baseline period [87] Change in symptom severity from previous report [78]	245 31,700 116 34	Every 90 days Daily Twice a day Weekly
	Binary outcome	Variable indicated if a report is followed by exacerbation event [80] Occurrence of symptom in any day of a time window [85]	2,374 182,991	Daily Daily (3 days)
		Dichotomised score one week following the prediction date [104]	210	Weekly
	Feature for each timeline	Score added as an input feature at every measurement [82] Created a timeline of best overall responses (BORs) [103]	83 31	Weekly Weekly
Not considered	Model for each timeline	Treated the 2- and 8-week measures as if assessed at baseline [81] Three models that used 7, 14, and 21 days as inputs [116]	1,003 20	3 time-points 3 time-points
	Selected 1 value for analysis	If patient had multiple follow-up events, the first was chosen [77] The assessment with the highest overall score was used [79]	494 11,761	Irregular^a^ Bi-weekly
		Score was updated at each assessment [86]	212,615	Irregular^a^

^a^Irregular measurements indicate that the reports were completed at any clinical event that occurred

Three papers which used time-series data did not report how the temporality was addressed [83, 109, 110], and are not included in this table

### Model evaluation

The evaluation metrics varied across the studies. Area under the curve (AUC) was most commonly used (*n* = 60, 64%), and 32 (53%) of papers used this value to assess model performance with imbalanced classes. Other frequently used performance metrics were recall, also known as sensitivity (*n* = 44, 47%), accuracy (*n* = 43, 46%), and specificity (*n* = 34, 36%). The majority of the studies used multiple performance metrics (*n* = 83, 88%). Variable importance analysis was performed by 64 studies (68%) 61 of which (95%) reported PROMs data being valuable for prediction. Seventy-nine papers (84%) provided information on the best performing model. Regression models were the most frequently selected as best-performing algorithms (*n* = 24, 30%), followed by boosting methods (*n* = 20, 25%), random forest (*n* = 10, 13%) and neural network (*n* = 10, 13%).

### Clinical relevance and adoption

No studies reported that the developed methods had been applied in the clinical practice. Although we acknowledge, that in such multidisciplinary research clinicians are generally involved in the study design, only 3 papers (3%) explicitly mentioned how clinicians contributed to the model development. They helped selecting input variables [30, 111], or creating a testing set [100]. No papers mentioned patients involvement in the model development or any part of the study design. The majority of papers reported age (*n* = 63, 67%) and gender (*n* = 60, 64%) of study participants and only 24 (26%) studies reported ethnicity. Papers were classified into 3 different categories, inspired by a previously conducted scoping review [118]: internal validation (one source of data used for training and validation, including cross-validation or holdout sample for validation on unseen data from the same dataset), external validation (the model developed on one dataset and then tested/validated on a completely new (i.e. external) dataset) or deployment (”integrated into a prototype application, and evaluated for its feasibility in clinical workflows”[118]). Based on these definitions, 81 papers (86%) were in the internal validation stage, 10 (11%) completed external validation, and 3 papers (3%) were in the deployment stage.

## Discussion

This scoping review aimed to identify AI methods used on PROMs data to predict patient outcomes. The analysis of 94 papers allowed the exploration of algorithms applied on complex patient-reported data and revealed the opportunities, challenges and best practice recommendations for AI medical research involving PROMs. The main findings suggest the variety of data types and evaluation metrics used, as well as inconsistencies in data pre-processing and model development design and reporting.

### Study characteristics

Due to fragmented data collection of PROMs, incorporating them into AI systems is very challenging [119]. Therefore, the majority of papers in this study have small sample size. In orthopedics settings PROMs have been increasingly collected as a part of routine care [120], which explains the large proportion of orthopedics papers with medium-to-large datasets. The large sample size was common in mental health papers, as mental health screening and diagnostic tools are usually based on PROMs, and there is a long-standing history of using such tools [121]. Contrary to orthopedics and mental health settings, PROMs collection in other healthcare domains is very limited. The respiratory datasets were mainly very small or very large. The papers with very large sample size were predicting outcomes related to COVID- 19 pandemic, where mobile applications collecting PROMs became more common [122]. Most of the studies analysed data collected specifically for research studies, rather than in clinical practice, which might introduce biases related to inclusion and exclusion criteria. Due to inconsistent PROMs questionnaires used across different studies, the comparison of results is limited. Therefore, using standardised and validated measures can help explore the overall predictive value of PROMs. The peak in the use of AI methods for all domains was between 2021 and 2022. This recent increase is compatible with a scoping review on AI in healthcare, where 71% of studies were published between 2020 and 2022 [123].

### Data pre-processing

Missing data in AI research is an important aspect to investigate, as it can lead to various biases [124]. Therefore, the inconsistencies in reporting data quality in the analysed studies are concerning. The justification of using data imputation techniques was also poor, whilst most commonly used techniques (complete case analysis and mean/median/mode imputations) can frequently cause bias [124]. Only a small number of papers used KNN-based imputation, which can reach the accuracy of complete data with a low performance difference [125]. Studies applying AI methods on PROMs should ensure that missing data are reported and any imputation methods are justified [124]. Another inconsistency between the papers was caused by various methods for handling class imbalance in classification tasks. Papers which reported class imbalance often did not attempt to balance the data, which prevents models from appropriate learning from the training set. Furthermore, most of these papers used AUC as a performance metric, which require the balanced setting to avoid bias [126]. The papers which reported balancing data have also done it inconsistently and without justification. Balancing data prior to train and test split can cause issues in model validation, as real data is never perfectly balanced. Therefore, it is important to evaluate model performance on test set unaffected by sampling methods [127]. The choice of performance metrics should also be justified and able to uncover potential bias caused by class imbalance (for example balanced accuracy and F1 score, instead of accuracy and AUC).

### Model development

The studies in this review reported model development process inconsistently, with majority of studies missing model hyperparameters reporting. According to Jha et al. (2023)[128], it should be”the ethical obligation” to document all stages of model development that are essential for the reproducibility of results. Therefore, model hyperparameters and their optimisation technique should always be reported and justified. The missingness in data was also handled and reported inconsistently, which is an important step for reproducibility as well. The lack of large PROMs datasets prevents applying deep learning methods, which can be extremely useful in capturing patterns in high-dimensional data or dependencies that other algorithms can’t capture [129]. Only the simple”vanilla” neural network was applied more often than some of the basic ML models. Studies which collected data in multiple timepoints often did not address the temporality at all or analysed time-series data through data pre-processing strategies and conventional ML models. Only 3 papers used DL models that are appropriate for temporal processing. These are for example LSTM or GRU methods. Most papers in this review chose various ways to include temporal information through feature engineering, as described in Table 3. Nevertheless, DL models have been more successful in accurate predictions of patients’ outcomes when applied to time-series data, as they are able to process more complex dependencies in high dimensionality and temporality of medical data [130].

### Model evaluation

Most studies used multiple evaluation metrics, which allow between-studies comparisons and in-depth analysis of model performance. Variable importance analysis was also commonly conducted, which supports the explainability of AI models [131]. Furthermore, the studies used multiple models, which allowed them to select the best-performing one. The analysis of these showed that most common models were rarely selected as best models (e.g., random forest was selected as the best model only 20% of the times). However, voting classifier was used only 4 times, but selected as the best model 3 times. This suggest that further studies should perhaps pay more attention to models that are used less-frequently, which have a potential to perform better.

### Clinical relevance and adoption

Limited reporting of clinician and patient engagement in the study development is of concern. This process is known as crucial for ensuring a patient-centred research and feasibility of the studies [132]. Involving stakeholders also can help building trust of the public to AI researchers, and as a result support the implementation of the studied tools in clinical practice [133]. Another issue arising from this review is the lack of external validation of the model performance in the studies, which is an essential step to potential clinical adoption. Assessing the model performance in a different setting may show different model performance which might suggest bias in the original study [118]. This review shows that new models keep being developed to address original problems, without taking the studies further and exploring their potential in the real-world settings. Therefore, validating existing models on external datasets and communicating the design and results with stakeholders should be the next step to support the adoption of AI methods in clinical practice. Furthermore, the majority of papers did not provide any information on the ethnicity of the study participants. Ensuring diverse study population is an essential ethical consideration and lack of ethnic information can further contribute to deepening healthcare inequalities [128].

### Strengths and limitations

The main strength of this review is that it identifies AI models applied on self-reported data for predicting patient outcomes in all healthcare domains. The use of 6 different databases from both health and computer science field helped reaching many relevant papers, which might have been omitted by reviews using limited number of databases. This paper also analyses the rigour of model development and evaluation reporting. It focuses on clinical adoption potential, from the perspective of patients and clinicians involvement and ethical consideration of participants’ diversity. The limitations of this study include the possibility of omitting the studies published in language other than English. Only published studies were considered, which might affect the conclusions and further deepen the publication bias. Since the study focuses on rigour in model development, evaluation, and wider stakeholder engagement, it is important to note, that the results are based on what was reported, and not what was done in the included papers.

## Conclusions

The analysis of 94 papers in this scoping review revealed the potential of using PROMs data in AI healthcare research, and inconsistencies in conducting and reporting these studies. It showed the importance of justification of chosen data pre-processing and model development methods, and the involvement of all stakeholders during the study. Our future work will involve applying AI on PROMs data and further explore the potential of time-series patient-reported data for healthcare outcomes predictions. We believe that insights from this paper can inform the rigorous implementation of AI models in clinical practice.

## Supplementary Information

Supplementary Material 1. Figure 1: PRISMA checklist for scoping reviews part 1. Figure 2: PRISMA checklist for scoping reviews part 2. Table 1: Search strategy. Table 2: Study characteristics and pre-processing methods used by studies included in the review. Table 3: Model development and evaluation, including study characteristics of papers included in the review.

## Data Availability

No datasets were generated or analysed during the current study.
