# Supplementary material for: Using artificial intelligence to predict patient outcomes from patient-reported outcome measures: a scoping review
Source: Health Qual Life Outcomes. 2025 Apr 11;23:37. doi: 10.1186/s12955-025-02365-z (PMC11987430; doi:10.1186/s12955-025-02365-z)
Supplement: Supplementary file 1 — Supplementary Material 1. Figure 1: PRISMA checklist for scoping reviews part 1. Figure 2: PRISMA checklist for scoping reviews part 2. Table 1: Search strategy. Table 2: Study characteristics and pre-processing methods used by studies included in the review. Table 3: Model development and evaluation, including study characteristics of papers included in the review. [file 12955_2025_2365_MOESM1_ESM.docx]

Figure 1: PRISMA checklist for scoping reviews part 1. Figure 2: PRISMA checklist for scoping reviews part 2.

Table 1: Search strategy. Table 2: Study characteristics and pre-processing methods used by studies included in the review. Table 3: Model development and evaluation, including study characteristics of papers included in the review

Supplementary Materials


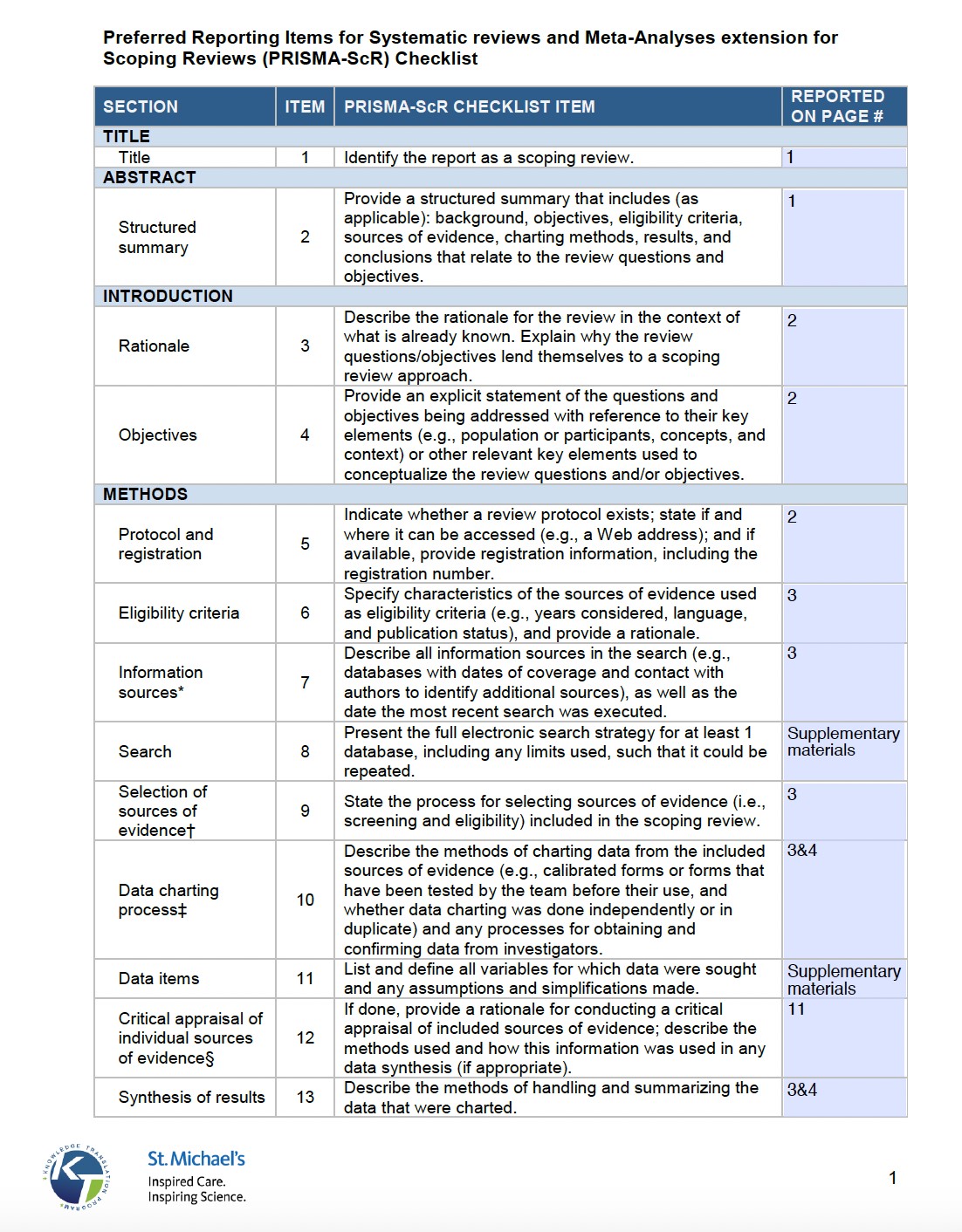


Figure 1: PRISMA checklist for scoping reviews part 1.


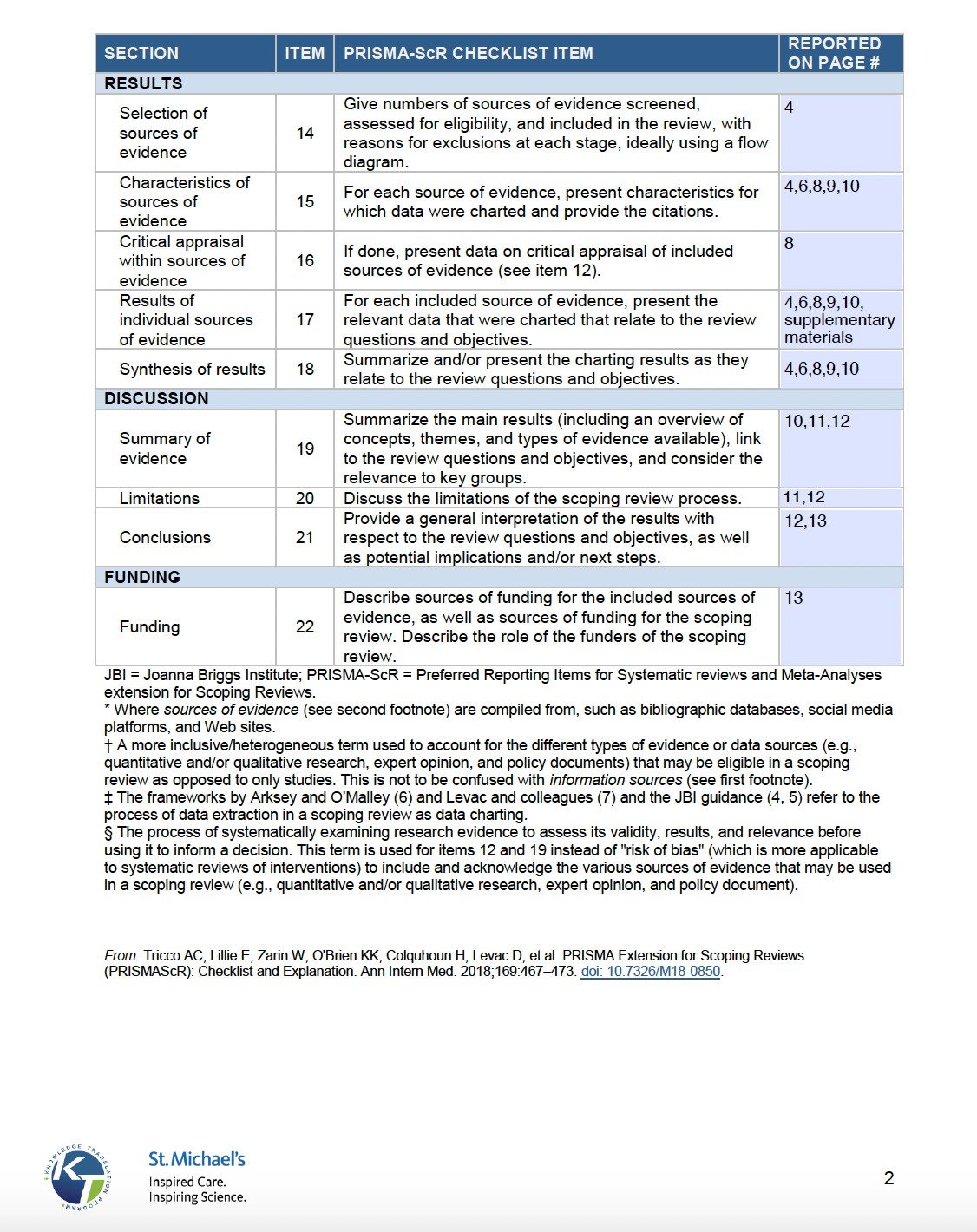


Figure 2: PRISMA checklist for scoping reviews part 2.

Table 1: Search strategy

| **Database** | **Search strategy** |
| --- | --- |
| Web of Science, IEEE Xplore, ACM Digital Library | “artificial intelligence” OR “AI” OR “machine learning” OR “deep learning” OR “neural network” OR “neural networks” OR algorithm* OR “rule-based” OR “rule based” AND “predictive model” OR “predictive models” OR “predictive modelling” OR “predictive modeling” OR predict* OR forecast* OR prognos* OR “predicting outcomes” AND “symptom tracking” OR “symptom-tracking” OR “symptom tracker” OR “symptom trackers” OR “symptom assessment” OR “symptom assessing” OR “symptom-assessing” OR “symptom report” OR “symptom reports” OR “symptom reporting” OR “symptom-reporting” OR “symptom monitoring” OR “symptom-monitoring” OR “patient-reported symptom” OR “patient-reported symptoms” OR “patient reported symptom” OR “patient reported symptoms” OR “self-reported symptom” OR “self-reported symptoms” OR “self reported symptom” OR “self reported symptoms” OR “patient-reported outcome” OR “patientreported outcomes” OR “patient reported outcome” OR “patient reported outcomes” OR “patient-reported outcome measure” OR “patient-reported outcome measures” OR “patient reported outcome measure” OR “patient reported outcome measures” OR “PROMs” OR “self-reported outcome” OR “self-reported outcomes” OR “self reported outcome” OR “self reported outcomes” OR “patient-reported side effect” OR “patient reported side effect” OR “patient reported side effects” OR “patient-reported side effects” OR “self-reported side effect” OR “self reported side effect” OR “self reported side effects” OR “self-reported side effects” |
| Cochrane Central Register of Controlled Trials | “artificial intelligence” OR “AI” OR “machine learning” OR “deep learning” OR “neural network” OR “neural networks” OR algorithm* OR “rule-based” OR “rule based” AND “predictive model” OR “predictive models” OR “predictive modelling” OR “predictive modeling” OR predict* OR forecast* OR prognos* OR “predicting outcomes” AND “symptom tracking” OR “symptom-tracking” OR “symptom tracker” OR “symptom trackers” OR “symptom assessment” OR “symptom assessing” OR “symptom-assessing” OR “symptom report” OR “symptom reports” OR “symptom reporting” OR “symptom-reporting” OR “symptom monitoring” OR “symptom-monitoring” OR “patient reported symptom” OR “patient reported symptoms” OR “self reported symptom” OR “self reported symptoms” OR “patient reported outcome” OR “patient reported outcomes” OR “patient reported outcome measure” OR “patient reported outcome measures” OR “PROMs” OR “self reported outcome” OR “self reported outcomes” OR “patient reported side effect” OR “patient reported side effects” OR “self reported side effect” OR “self reported side effects” |
| Medline | exp Artificial Intelligence/ OR AI.mp. OR exp Machine Learning/ OR exp Deep Learning/ OR exp Neural Networks, Computer/ OR exp Algorithms/ OR algorithm*.mp. OR “rule-based”.mp. OR “rule based”.mp AND “predictive model”.mp. OR “predictive models”.mp. OR “predictive modelling”.mp. OR “predictive modeling”.mp. OR predict*.mp. OR forecast*.mp. OR prognos*.mp. OR “predicting outcomes”.mp. AND (symptom adj2 (track* or -track* or assess* or assess* or report* or -report* or monitor* or -monitor*)).mp. OR “patient-reported symptom”.mp. OR “patient-reported symptoms”.mp. OR “self-reported symptom”.mp. OR “self-reported symptoms”.mp. OR exp Patient Reported Outcome Measures/ OR “patient-reported outcome measure”.mp. OR “patient-reported outcome measures”.mp. OR “patient-reported outcome”.mp. OR “patient-reported outcomes”.mp. OR PROMs.mp. OR “self-reported outcome”.mp. OR “selfreported outcomes”.mp. OR “patient-reported side effect”.mp. OR “patientreported side effects”.mp. OR “self-reported side effect”.mp. OR “self-reported side effects”.mp. |
| Embase | exp artificial intelligence/ OR AI.mp. OR exp machine learning/ OR exp deep learning/ OR exp artificial neural network/ OR “neural networks”.mp. OR exp algorithm/ OR algorithm*.mp. OR “rule-based”.mp. AND exp predictive model/ OR “predictive models”.mp. OR “predictive modelling”.mp. OR “predictive modeling”.mp. OR predict*.mp. OR forecast*.mp. OR prognos*.mp. OR “predicting outcomes”.mp. AND (symptom adj2 (track* or -track* or assess* or -assess* or report* or -report* or monitor* or -monitor*)).mp. OR “patient-reported symptom”.mp. OR “patient-reported symptoms”.mp. OR “self-reported symptom”.mp. OR “self-reported symptoms”.mp. OR exp patient reported outcome/ OR “patientreported outcomes”.mp. OR “self-reported outcome”.mp. OR “self-reported outcomes”.mp. OR “patient-reported side effect”.mp. OR “patient-reported side effects”.mp. OR “patient-reported outcome measure”.mp. OR “patient-reported outcome measures”.mp. OR PROMs.mp. OR “self-reported side effect”.mp. OR  “self-reported side effects”.mp. |

Table 2: Study characteristics and pre-processing methods used by studies included in the review.

| Authors | Year | Health domain | Sample size | Was outcome self-reported? | Imbalanced data acknowledged? | Need for balancing reported? | Whats the class distribution? | Upsampling type | Missingness reported? |
| --- | --- | --- | --- | --- | --- | --- | --- | --- | --- |
| Klemt et al [29] | 2022 | orthopedics | 4526 | yes | no | no | not reported | n/a | no |
| Sandham et al [107] | 2022 | palliative care | 751 | no | yes | no | n/a | imbalanced | yes |
| Tennenhouse et al [30] | 2020 | mental health | 637 | no | no | no | imbalanced | n/a | yes |
| Zhang et al [31] | 2021 | orthopedics | 1508 | yes | yes | yes | n/a | partially balanced | yes |
| Zhang et al [32] | 2022 | orthopedics | 2840 | yes | yes | yes | n/a | partially balanced | yes |
| Haeberle et al [103] | 2021 | orthopedics | 3147 | no | yes | yes | n/a | testing data | yes |
| Verma et al [33] | 2021 | orthopedics | 218 | yes | no | no | no classification | n/a | no |
| Buus et al [34] | 2022 | orthopedics | 201 | yes | no | no | balanced | n/a | yes |
| Staartjes et al [35] | 2019 | orthopedics | 422 | MCID | yes | yes | n/a | balanced | yes |
| Huber et al [36] | 2019 | orthopedics | 34,110 | yes | yes | no | n/a | partially balanced | yes |
| Wang et al [26] | 2021 | oncology | 823 | yes | no | no | no classification | n/a | yes |
| Xu et al [37] | 2022 | oncology | 630 | no | no | no | balanced | n/a | yes |
| Verma et al [88] | 2022 | orthopedics | 1040 | combined | yes | no | n/a | imbalanced | no |
| Harris et al [38] | 2020 | orthopedics | 587 | MCID | no | no | not reported | n/a | no |
| Siccoli et al [39] | 2019 | orthopedics | 635 | MCID | yes | yes | n/a | partially balanced | yes |
| Pfob et al [40] | 2021 | oncology | 1553 | combined | yes | no | n/a | imbalanced | yes |
| Curtis et al [76] | 2022 | orthopedics | 494 | no | no | no | imbalanced | n/a | yes |
| Iivanainen et al [77] | 2021 | oncology | 34 | yes | yes | no | n/a | imbalanced | no |
| Pedersen et al [41] | 2022 | orthopedics | 1968 | MCID | yes | yes | n/a | balanced | yes |
| Katakam et al [42] | 2022 | orthopedics | 744 | MCID | no | no | imbalanced | n/a | yes |
| Crowson et al [89] | 2020 | hearing | 1604 | yes | no | no | imbalanced | n/a | yes |
| Josephson et al [43] | 2021 | neurology | 201 | yes | no | no | imbalanced | n/a | yes |
| Ramkumar et al [44] | 2021 | orthopedics | 153 | MCID | no | no | imbalanced | n/a | no |
| Noel et al [78] | 2022 | oncology | 11761 | yes | yes | no | n/a | imbalanced | yes |
| Munn et al [45] | 2022 | orthopedics | 1432 | yes | no | no | imbalanced | n/a | yes |
| Kober et al [46] | 2021 | oncology | 1217 | yes | no | no | not reported | n/a | yes |
| Durand et al [47] | 2020 | orthopedics | 1503 | no | no | no | imbalanced | n/a | yes |
| Harrison et al [48] | 2022 | orthopedics | 1916 | yes | no | no | balanced | n/a | yes |
| Kumar et al [49] | 2020 | orthopedics | 2887 | yes | no | no | balanced | n/a | yes |
| Wshah et al [50] | 2019 | mental health | 90 | yes | no | no | not reported | n/a | yes |
| Lu et al [51] | 2022 | orthopedics | 381 | yes | no | no | imbalanced | n/a | yes |
| Kunze et al [64] | 2021 | orthopedics | 818 | MCID | no | no | imbalanced | n/a | yes |
| Kalweit et al [27] | 2021 | orthopedics | 9500 | yes | no | no | not reported | n/a | yes |
| Milella et al [90] | 2022 | orthopedics | 6521 | MCID | yes | yes | n/a | partially balanced | yes |
| Loos et al [53] | 2022 | orthopedics | 2224 | MCID | yes | yes | n/a | balanced | no |
| Hsu et al [109] | 2022 | neurology | 3495 | no | no | no | imbalanced | n/a | no |
| Iivanainen et al [101] | 2022 | oncology | 31 | no | yes | no | n/a | imbalanced | no |
| Polce et al [91] | 2021 | orthopedics | 331 | yes | no | no | imbalanced | n/a | yes |
| Chmiel et al [79] | 2022 | respiratory | 2374 | yes | no | no | imbalanced | n/a | yes |
| Sun et al [104] | 2021 | orthopedics | 1,198 | no | yes | yes | n/a | partially balanced | yes |
| Xuyi et al [132] | 2021 | oncology | 46,104 | yes | no | no | not reported | n/a | yes |
| Shipston-Sharman et al [54] | 2022 | neurology | 2581 | yes | no | no | imbalanced | n/a | no |
| Long et al [111] | 2017 | orthopedics | 88 | no | no | no | imbalanced | n/a | no |
| Luo et al [102] | 2015 | respiratory | 210 | yes | yes | yes | n/a | partially balanced | no |
| Ziobrowski et al [80] | 2021 | mental health | 1003 | yes | no | no | imbalanced | n/a | yes |
| Tan et al [55] | 2013 | respiratory | 72 | no | no | no | balanced | n/a | yes |
| Miranda et al [110] | 2021 | respiratory | 217,580 | no | yes | yes | n/a | partially balanced | no |
| Annapureddy et al [81] | 2020 | mental health | 83 | yes | yes | yes | n/a | partially balanced | yes |
| Thanathamathee [92] | 2014 | mental health | 3115 | yes | no | no | imbalanced | n/a | no |
| Mendoza et al [93] | 2022 | respiratory | 1434868 | no | yes | yes | n/a | balanced | no |
| Rahman et al [56] | 2022 | mental health | 775 | yes | no | no | not reported | n/a | no |
| Wang et al [133] | 2010 | sub-health state | 572 | no | yes | no | n/a | imbalanced | yes |
| Lee [57] | 2017 | mental health | 9,089 | no | no | no | imbalanced | n/a | yes |
| Xu et al [94] | 2022 | mental health | 25214 | yes | no | no | not reported | n/a | no |
| Zhong et al [82] | 2022 | mental health | 915 | yes | yes | no | n/a | imbalanced | yes |
| Pfob et al [134] | 2022 | oncology | 1921 | yes | no | no | imbalanced | n/a | yes |
| Van der Stap [115] | 2022 | oncology | 532 | yes | no | no | no classification | n/a | yes |
| Sidey-Gibbons et al [83] | 2022 | oncology | 245 | no | yes | yes | n/a | partially balanced | yes |
| Nowinka et al [58] | 2022 | orthopedics | 5947 | yes | yes | yes | n/a | imbalanced | yes |
| Arkin et al [59] | 2020 | oncology | 189 | no | no | no | imbalanced | n/a | no |
| Fu et al [95] | 2018 | oncology | 355 | no | no | no | balanced | n/a | no |
| Schultebraucks et al [105] | 2021 | mental health | 473 | yes | yes | yes | n/a | balanced | yes |
| Hoogendam et al [60] | 2022 | orthopedics | 2119 | MCID | yes | yes | n/a | balanced | yes |
| Camp et al [61] | 2022 | neurology | 453 | MCID | yes | no | n/a | imbalanced | yes |
| Canas et al [84] | 2021 | respiratory | 182991 | no | yes | no | n/a | imbalanced | no |
| Wardenaar [62] | 2021 | mental health | 2,981 | yes | no | no | imbalanced | n/a | yes |
| Bugajski [114] | 2021 | respiratory | 20 | yes | no | no | not reported | n/a | no |
| Sim et al [63] | 2020 | oncology | 809 | no | yes | yes | n/a | balanced | yes |
| Pan et al [96] | 2020 | oncology | 86 | yes | no | no | imbalanced | n/a | yes |
| Kunze et al [52] | 2020 | orthopedics | 616 | MCID | no | no | imbalanced | n/a | yes |
| Sutradhar [85] | 2020 | oncology | 212615 | no | no | no | not reported | n/a | no |
| Verma et al[135] | 2022 | orthopedics | 293 | yes | no | no | no classification | n/a | no |
| Smith et al [65] | 2022 | mental health | 361 | yes | no | no | no classification | n/a | yes |
| Agochukwu-Mmonu et al [97] | 2022 | oncology | 2653 | yes | no | no | imbalanced | n/a | yes |
| Goldstein et al [86] | 2021 | respiratory | 116 | no | yes | yes | n/a | balanced | no |
| Bone et al [66] | 2021 | mental health | 2317 | yes | no | no | balanced | n/a | yes |
| O’Driscoll et al [67] | 2021 | mental health | 2858 | yes | no | no | not reported | n/a | yes |
| Tschuggnall et al [106] | 2021 | orthopedics | 1047 | combined | no | no | not reported | n/a | yes |
| Rajpurkar et al [2] | 2020 | mental health | 518 | yes | no | no | not reported | n/a | no |
| Shahzad et al [113] | 2020 | mental health | 600 | yes | no | no | imbalanced | n/a | no |
| Shi et al [68] | 2012 | oncology | 402 | yes | no | no | imbalanced | n/a | no |
| Pua et al [69] | 2020 | orthopedics | 4026 | yes | no | no | imbalanced | n/a | yes |
| Chekroud et al [98] | 2016 | mental health | 4041 | yes | no | no | not reported | n/a | yes |
| Nijeweme-D’Hollosy et al[99] | 2018 | orthopedics | 1288 | no | no | no | imbalanced | n/a | no |
| Rahman et al [87] | 2019 | orthopedics | 31,700 | yes | yes | yes | n/a | balanced | no |
| Langenberger et al [75] | 2022 | orthopedics | 1,843 | MCID | yes | no | n/a | n/a | yes |
| Pappot et al [108] | 2023 | oncology | 238 | no | no | no | imbalanced | n/a | yes |
| Hasannejadasl et al [71] | 2023 | oncology | 964 | yes | yes | yes | n/a | partially balanced | yes |
| Xu et al [72] | 2023 | oncology | 3,058 | yes | yes | no | n/a | n/a | yes |
| Grant et al [28] | 2023 | oncology | 105,129 | no | no | no | balanced | n/a | yes |
| Tian et al [73] | 2023 | cardiovascular | 941 | no | yes | yes | n/a | partially balanced | yes |
| Park et al [74] | 2023 | orthopedics | 1,141 | MCID | no | no | balanced | n/a | yes |
| Goldstein et al [100] | 2023 | endometriosis | 886 | no | no | no | balanced | n/a | no |
| Langenberger [70] | 2023 | orthopedics | 5,934 | no | no | no | balanced | n/a | yes |

Table 3: Model development and evaluation, including study characteristics of papers included in the review.

| Authors | Hyperparameter tuning | Hyperparameters reported? | Variable importance reported? | Best performing model | Clinicians involvement? | Country | Stage of development | Age reported? | Gender reported? | Ethnicity reported? |
| --- | --- | --- | --- | --- | --- | --- | --- | --- | --- | --- |
| Klemt et al [29] | nr | no | yes | NN | no | USA | internal validation | yes | yes | no |
| Sandham et al [107] | experimentally selected | no | yes | NB | no | New Zealand | internal validation | no | yes | yes |
| Tennenhouse et al [30] | done, no technique | no | yes | regression | yes | Canada | internal validation | yes | yes | yes |
| Zhang et al [31] | cross-validation | no | yes | regression | no | Singapore | internal validation | yes | yes | no |
| Zhang et al [32] | cross-validation | no | yes | regression | no | Singapore | internal validation | yes | yes | yes |
| Haeberle et al [103] | nr | no | yes | n/a | no | USA | internal validation | yes | yes | yes |
| Verma et al [33] | grid search | no | yes | regression | no | Norway | internal validation | no | no | no |
| Buus et al [34] | grid search | no | no | RF | no | Denmark | internal validation | yes | yes | no |
| Staartjes et al [35] | grid search | no | yes | NN | no | Netherlands | internal validation | yes | yes | no |
| Huber et al [36] | grid search | no | yes | boosting | no | Germany | internal validation | yes | yes | no |
| Wang et al [26] | nr | no | no | LSTM | no | USA | internal validation | no | no | no |
| Xu et al [37] | Bayesian Optimisation | yes | yes | boosting | no | USA | internal validation | yes | yes | yes |
| Verma et al [88] | random search | yes | yes | regression | no | Netherlands | internal validation | no | no | no |
| Harris et al[38] | nr | no | no | QDA | no | USA | external validation | yes | yes | yes |
| Siccoli et al [39] | done, no technique | no | yes | nr | no | Netherlands | internal validation | yes | yes | no |
| Pfob et al [40] | grid search | yes | yes | NN | no | USA and Canada | internal validation | yes | no | yes |
| Curtis et al [76] | random search | no | yes | RF | no | USA | internal validation | yes | yes | yes |
| Iivanainen et al[77] | grid search | no | yes | boosting | no | Finland | internal validation | no | no | no |
| Pedersen et al[41] | done, no technique | no | no | SVM | no | Danemark | internal validation | yes | yes | no |
| Katakam et al [42] | nr | no | yes | regression | no | USA | internal validation | yes | yes | no |
| Crowson et al[89] | nr | no | yes | NN | no | Canada | internal validation | yes | yes | no |
| Josephson et al [43] | nr | no | no | nc | no | Canada and Sweden | internal validation | yes | yes | no |
| Ramkumar et al [44] | nr | no | yes | NB | no | USA | internal validation | yes | no | no |
| Noel et al [78] | grid search | no | yes | boosting | no | USA | internal validation | yes | yes | no |
| Munn et al [45] | nr | no | yes | regression | no | Canada | internal validation | yes | yeas | no |
| Kober et al [46] | nr | no | yes | RF | no | USA | internal validation | yes | yes | yes |
| Durand et al [47] | grid search | no | yes | SVM | no | USA | internal validation | yes | yes | no |
| Harrison et al[48] | nr | no | no | regression | no | Scotland | internal validation | yes | yes | no |
| Kumar et al [49] | nr | no | yes | Wide and deep | no | USA | internal validation | yes | yes | no |
| Wshah et al [50] | grid search | yes | yes | SVM | no | UK | internal validation | yes | yes | yes |
| Lu et al [51] | nr | no | yes | RF | no | USA | internal validation | yes | yes | no |
| Kunze et al [64] | nr | no | yes | boosting | no | USA | internal validation | yes | yes | yes |
| Kalweit et al [27] | random search | yes | yes | AdaptiveNet | no | Switzerland | internal validation | yes | no | no |
| Milella et al [90] | random search | yes | no | boosting | no | Italy | internal validation | yes | yes | no |
| Loos et al [53] | nr | no | no | boosting | no | Netherlands | internal validation | yes | yes | no |
| Hsu et al [109] | nr | no | yes | SVM | yes | Taiwan | internal validation | yes | yes | no |
| Iivanainen et al[101] | nr | no | yes | boosting | no | Finland | internal validation | yes | yes | no |
| Polce et al [91] | nr | no | yes | SVM | no | USA | internal validation | yes | yes | no |
| Chmiel et al [79] | Bayesian Optimisation | no | yes | RF | no | UK | internal validation | yes | yes | no |
| Sun et al [104] | nr | yes | yes | n/a | no | USA | internal validation | yes | yes | yes |
| Xuyi et al [132] | nr | yes | no | n/a | no | Canada | internal validation | yes | yes | no |
| Shipston-Sharman et al [54] | experimentally selected | yes | no | regression | no | Scotland | internal validation | yes | yes | no |
| Long et al [111] | cross-validation | no | yes | nc | no | UK | internal validation | yes | yes | no |
| Luo et al [102] | done, no technique | no | no | boosting | no | USA | internal validation | yes | yes | yes |
| Ziobrowski et al [80] | super learner | no | yes | nc | no | USA | internal validation | yes | yes | yes |
| Tan et al [55] | nr | no | yes | n/a | no | USA | internal validation | no | no | no |
| Miranda et al [110] | cross-validation | yes | no | NN | no | Brazil | internal validation | yes | yes | yes |
| Annapureddy et al [81] | Bayesian Optimisation | no | no | voting classifier | no | USA | internal validation | no | yes | no |
| Thanathamathee [92] | nr | no | yes | boosting | no | Thailand | internal validation | no | no | no |
| Mendoza et al [93] | nr | no | yes | boosting | no | Philippines | internal validation | no | no | no |
| Rahman et al[56] | random search | no | yes | regression | no | Jordan | internal validation | no | no | no |
| Wang et al[133] | nr | no | yes | n/a | no | China | internal validation | no | no | no |
| Lee [57] | nr | no | yes | regression | no | Korea | internal validation | yes | yes | no |
| Xu et al[94] | nr | yes | yes | voting classifier | no | China | internal validation | no | no | no |
| Zhong et al [82] | grid search | yes | yes | boosting | no | Sweden | internal validation | no | no | no |
| Pfob et al [134] | nr | no | yes | regression | no | USA and Canada | internal validation | yes | yes | yes |
| Van der Stap [115] | nr | no | no | n/a | no | Netherlands | internal validation | yes | yes | no |
| Sidey-Gibbons et al [83] | grid search | yes | yes | NN | no | USA | internal validation | yes | yes | yes |
| Nowinka et al [58] | done, no technique | yes | yes | regression | no | USA | external validation | yes | yes | yes |
| Arkin et al [59] | nr | no | yes | NN | no | Turkey | internal validation | yes | yes | no |
| Fu et al [95] | cross-validation | yes | no | NN | no | USA | internal validation | yes | yes | yes |
| Schultebraucks et al [105] | random search | yes | yes | SVM | no | USA | internal validation | yes? | ? | no |
| Hoogendam et al [60] | cross-validation | no | yes | regression | no | Netherlands | deployment | yes | yes | no |
| Camp et al[61] | grid search | no | yes | SVM | no | USA | deployment | yes | yes | yes |
| Canas et al [84] | nr | no | yes | hierarchical gaussian process | no | UK and USA | internal validation | yes | yes | no |
| Wardenaar[62] | nr | no | yes | SuperLearner | no | Netherlands | internal validation | yes | yes | no |
| Bugajski[114] | nr | yes | no | n/a | no | USA | internal validation | yes | yes | yes |
| Sim et al [63] | done, no technique | no | yes | bagging | no | South Korea | internal validation | yes? | ? | ? |
| Pan et al [96] | nr | no | no | boosting | no | USA | external validation | no | no | no |
| Kunze et al[52] | nr | no | yes | RF | no | USA | deployment | yes | no | no |
| Sutradhar[85] | nr | yes | no | NN | no | Canada | internal validation | yes | yes | no |
| Verma et al[135] | grid search | no | yes | boosting | no | Norway | external validation | no | no | no |
| Smith et al [65] | nr | no | no | regression | no | USA | external validation | yes | yes | yes |
| Agochukwu-Mmonu et al [97] | nr | no | yes | n/a | no | USA | internal validation | yes | yes | no |
| Goldstein et al [86] | nr | no | yes | RF | no | Israel | internal validation | yes | yes | no |
| Bone et al [66] | nr | yes | no | boosting | no | UK | external validation | yes | yes | no |
| O’Driscoll et al[67] | nr | yes | yes | regression | no | UK | internal validation | yes | yes | yes |
| Tschuggnall et al[106] | cross-validation | no | yes | RF | no | Austria | internal validation | no | no | no |
| Rajpurkar et al [2] | grid search | no | yes | n/a | no | UK and USA | internal validation | yes | yes | no |
| Shahzad et al [113] | experimentally selected | no | yes | n/a | no | Pakistan | internal validation | no | yes | no |
| Shi et al [68] | nr | yes | yes | NN | no | Taiwan | internal validation | yes | no | no |
| Pua et al [69] | cross-validation | no | yes | regression | no | Singapore | internal validation | yes | yes | yes |
| Chekroud et al [98] | cross-validation | no | yes | nc | no | USA | external validation | no | no | no |
| Nijeweme-D’Hollosy et al[99] | nr | no | yes | boosting | yes | Netherlands | internal validation | yes | no | no |
| Rahman et al[87] | nr | no | yes | regression | no | UK | internal validation | no | no | no |
| Langenberger et al[75] | cross-validation | yes | yes | RF, regression, boosting | no | Germany | internal validation | yes | yes | no |
| Pappot et al [108] | nr | yes | yes | random forest | no | Denmark | internal validation | no | yes | no |
| Hasannejadasl et al [71] | nr | no | yes | LR | no | Netherlands | external validation | yes | yes | no |
| Xu et al [72] | hypergrid search | yes | yes | LR | no | USA | external validation | no | no | no |
| Grant et al [28] | Bayesian Optimisation | no | yes | voting classifier | no | Canada | external validation | yes | yes | no |
| Tian et al [73] | search | yes | yes | XGBoost | no | China | internal validation | yes | yes | no |
| Park et al [74] | nr | yes | yes | regression | no | USA | internal validation | yes | yes | yes |
| Goldstein et al [100] | nr | no | yes | AdaBoost | no | Europe, US, Australia, Israel | internal validation | no | no | no |
| Langenberger [70] | grid search | no | yes | Xboost and regression | no | Germany | internal validation | yes | yes | no |
